# Learning from mistakes: Objective and subjective error magnitudes predict the testing effect

**DOI:** 10.3758/s13423-026-03009-z

**Published:** 2026-09-24

**Authors:** Aike Shi, Xiaonan L. Liu

**Affiliations:** 1 Department of Psychology, The Chinese University of Hong Kong, Hong Kong, Hong Kong; 2 Hong Kong Hub of Obstetric and Paediatric Excellence, The Chinese University of Hong Kong, Hong Kong, Hong Kong; 3 Brain and Mind Institute, The Chinese University of Hong Kong, Hong Kong, Hong Kong

**Keywords:** Testing effect, Retrieval practice, Error-driven learning, Metamemory, Confidence

## Abstract

The testing effect—where retrieval practice enhances memory more than restudy—remains mechanistically debated. We advance an error-driven learning (EDL) account, proposing that testing enhances memory by generating error signals that drive learning. Across two experiments using a color-wheel paradigm, we quantified retrieval errors on a continuous scale and examined three types of error signals: objective errors (OE), defined as the angular discrepancy between the correct color and the participant’s response; predicted errors (PredE), defined as participants’ prefeedback estimate of how inaccurate their response was; and perceived errors (PercE), defined as participants’ postfeedback estimate of the discrepancy between their response and the correct answer. Experiment 1 showed that larger OE during practice predicted greater memory updating. Experiment 2 further revealed that PredE and PercE uniquely predicted memory updating beyond OE. To formalize these dynamics, we developed a computational model (WRAP-E) that simulates distinct memory-strengthening roles for OE, PredE, and PercE across different stages of retrieval. Our findings highlight errors as not merely byproducts of failed recall, but as potent drivers of learning, both through their magnitude and metacognitive interpretation. This framework offers a unified account of the testing effect and carries implications for theories of memory, metacognition, and educational practice.

**Supplementary Information:**

The online version contains supplementary material available at https://doi.org/10.3758/s13423-026-03009-z.

## Introduction

A large number of studies have shown that testing not only assesses knowledge but also powerfully enhances memory (Adesope et al., 2017; Agarwal et al., 2008; Butler & Roediger, 2007; Jacoby et al., 2010; Pyc & Rawson, 2009, 2010). Specifically, learning materials followed by tests are typically remembered better than those followed by additional restudy opportunities. This robust phenomenon, termed the *testing effect* or the *retrieval practice effect*, has been replicated across a variety of materials, paradigms, and scenarios (Bjork et al., 2014; Butler & Roediger, 2007; Liu & Reder, 2016; Liu et al., 2018; Zheng et al., 2023).

Despite extensive empirical support, the mechanism underlying the testing effect is still under debate. Classic theories, such as the Transfer Appropriate Processing Account (Blaxton, 1989; Butler, 2010), the Release from Proactive Interference Account (Dang et al., 2021), the Episodic Context Account (Lehman et al., 2014), and the Semantic Elaboration Theory (Carpenter, 2009), can explain some findings, but struggle to generalize across findings (see Liu et al., 2021, for a review).

To address these limitations, a mechanistic model based on error-driven learning (EDL) has been proposed (Liu et al., 2021, 2024; Zheng et al., 2022). This account builds on Carrier and Pashler’s (1992) error-correction account of the testing effect, which proposed that retrieval attempts enhance learning by producing errors that can be corrected through feedback. Extending this idea, the EDL model proposes that error signals enhance memory by driving memory updating, with the magnitude of the updating proportional to the strength of the error signal. Consequently, tests strengthen memory more effectively than restudy by generating substantially larger error signals. Importantly, this does not imply that restudy is error-free. Errors can also arise during encoding or restudy when the presented target differs from the learner’s current memory representation (e.g., Greve et al., 2017). However, tests require learners to actively reconstruct the target, making the discrepancy between the retrieved response and the correct target more overt and typically larger than during passive reexposure. Supporting this account, prior studies have shown that learning from errors deepens encoding by driving stronger and more targeted representational change toward the correct patterns (e.g., Bahrick & Hall, 2005; Cyr & Anderson, 2012, 2015; Huelser & Metcalfe, 2012; Kane & Anderson, 1978; Wang & Yang, 2023; Yang et al., 2017). Moreover, committing errors prior to feedback has been shown to enhance the effectiveness of that feedback, leading to greater memory updating compared to receiving the same feedback without prior error generation (Potts & Shanks, 2014; Pupillo et al., 2023; Rumelhart et al., 1986; Shing et al., 2023).

A particularly illustrative example of error-potentiated learning comes from a classic study by Kornell et al. (2009), in which participants studied semantically related word pairs under conditions with or without guessing. In the guessing condition, participants were instructed to first guess the target word based on the cue word (e.g., factory–?) before seeing the correct cue-target pair (e.g., factory–plant). Despite all guesses being incorrect, recall performance improved, demonstrating that making errors can enhance memory, consistent with the EDL account.

However, such evidence provides only limited support for the EDL account because these studies typically rely on a binary manipulation of error signals, contrasting conditions with salient errors against conditions with smaller or less explicit errors (Kornell et al., 2009; Potts & Shanks, 2014). As a result, they do not test the core quantitative prediction of EDL, that is, larger errors should produce greater memory updating.

To more directly test the EDL mechanism, the present study moves beyond binary comparisons and examines whether the magnitude of retrieval errors predicts the magnitude of learning gains. Specifically, if EDL is correct, larger retrieval errors should elicit stronger learning signals, resulting in greater memory updating, reflecting a positive correlation between the error magnitude and the benefits of testing.

Furthermore, a more nuanced prediction of the EDL account is that the relationship between objective error (OE) magnitudes and learning should be modulated by subjective error perception. In other words, learners’ metacognitive interpretation and monitoring of errors may shape how effectively errors are used to update memory (Exton-McGuinness et al., 2015; Pupillo et al., 2023; Shing et al., 2023; Sinclair et al., 2021; Wang & Yang, 2023). Metamemory, which involves individuals’ ability to monitor and regulate their memory processes, can bias how OE are perceived. For example, people may misjudge the severity of errors based on their subjective confidence or familiarity during recall (Barenberg & Dutke, 2019; Jacoby et al., 2010). Specifically, when retrieval feels fluent and confident, people may underestimate the degree of error (Miller & Geraci, 2016; Murphy et al., 2022; Tullis et al., 2013). Conversely, difficult or uncertain retrieval can contribute to overestimation of the errors (Chen et al., 2019; Miller & Geraci, 2014; Zhang et al., 2019). Such overestimation may increase attention to potential errors, thereby amplifying the impact of OE when feedback is provided and resulting in stronger memory updating.

Additionally, another source of metacognitive judgment arises when individuals compare their recalled information to the actual studied content after feedback. This comparison helps them generate a more evidence-based estimation of errors and calibrate their memory more accurately even in the absence of the standard reference of OE.

In this paper, we differentiate between two types of metacognitive judgments of error: predicted error (PredE), which reflects the learner’s anticipatory estimate of possible error before feedback, and perceived error (PercE), which reflects the learner’s subjective estimate of the discrepancy after feedback. These subjective error signals are conceptually distinct from objective error (OE), which refers to the actual discrepancy between the retrieved response and the correct target. We hypothesize that PredE may shape error-driven learning by modulating how strongly OE is used when feedback is presented, whereas PercE may further guide memory updating during the subsequent postfeedback reencoding phase.

**Fig. 1:**
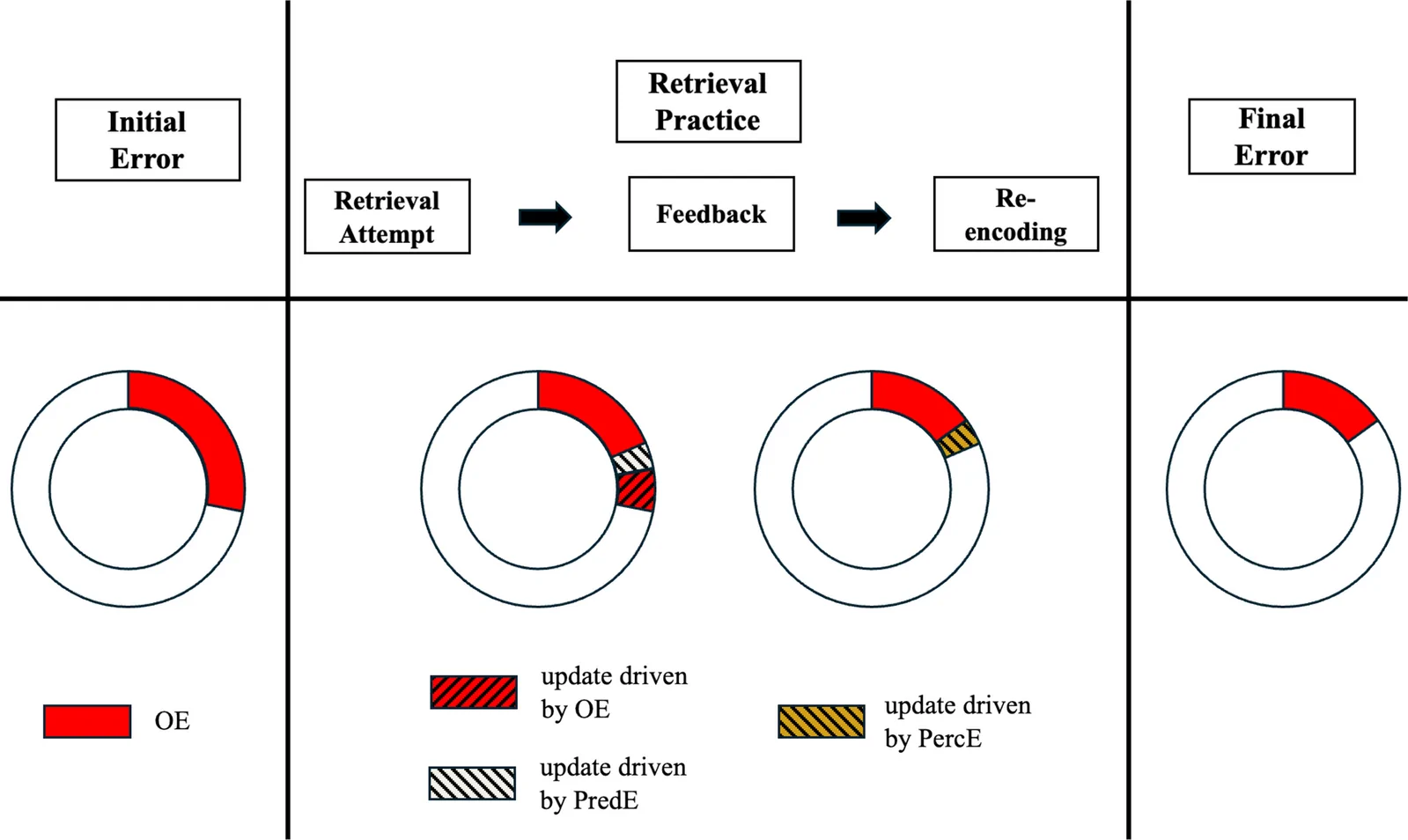
Illustration of the EDL account. The figure depicts changes in a memory representation on a color wheel during retrieval practice. The red region represents the initial objective error (OE), defined as the discrepancy between the learner’s current memory representation and the correct target. Retrieval practice involves two key stages: retrieval attempt and postretrieval reencoding. During the retrieval attempt stage, OE provides the primary discrepancy signal for memory updating, while predicted error (PredE; beige region) reflects the learner’s prefeedback expectation of error and may modulate the impact of OE. After feedback is provided, learners form a subjective assessment of the discrepancy, reflected in perceived error (PercE; gold region), which may further guide postretrieval reencoding. Together, these objective and subjective error signals drive memory updating, leading to reduced final error. (Color figure online)

Taken together, as illustrated in Fig. 1, the EDL model makes two key predictions:

1. The magnitude of errors positively predicts the extent of memory updating.
2. Learning from errors should arise from the joint and interactive contributions of OE, PredE, and PercE, with PredE modulating the impact of OE at feedback, while PercE further guiding memory updating during post-feedback reencoding.

To test these predictions, we employ a color wheel paradigm to quantify memory performance while measuring OE, PredE, and PercE across two experiments. This paradigm requires participants to memorize images with randomly assigned colors from a 360° continuous color circle (Oberauer et al., 2017; Sutterer & Awh, 2016). During recall, they reconstruct the studied color by selecting it from the wheel, with recall accuracy represented as a continuous value ranging from 0 to 180°, offering more precision than binary scoring.

Experiment 1 aims to replicate the typical testing effect and provide preliminary evidence supporting the EDL model. Experiment 2 extends the findings by examining how OE, PercE, PredE, and their interactions predict memory benefits from testing. In addition, we implement computational simulations using an adapted version of the dual-process working memory model (Liu et al., 2018; Popov & Reder, 2020; Zheng et al., 2023) to identify the functional stages at which different types of errors operate and to quantify their respective contributions to learning outcomes.

## Experiment 1

### Method

#### Participants

Thirty-three participants from the Chinese University of Hong Kong were recruited for the formal experiment (21 women, *M*_age_ = 21.08 years). All participants reported normal or corrected-to-normal vision and normal color vision, provided informed consent before the experiment and were compensated immediately after completion. The sample size was determined based on the expected testing effect comparison between Test and Restudy conditions, using a paired-sample design. A pilot study (*N* = 10) indicated that at least 28 participants were needed to detect the testing effect difference at *α* =.05 with 80% power. To ensure adequate power while allowing for potential exclusions, we recruited 33 participants. Because the main analyses used trial-level mixed-effects models, we also conducted a sensitivity analysis for the mixed-effects design (Murayama et al., 2022), which indicated that the current design had sufficient sensitivity to fixed effects of approximately Cohen’s *d* = 0.56 or larger, suggesting adequate sensitivity for moderate effects in the mixed-effects models (Cohen, 1988). This effect size is consistent with the typical magnitude of the testing effect reported in existing meta-analyses (Adesope et al., 2017; Rowland, 2014; Yang et al., 2021). Four participants were excluded from the formal analyses due to extremely high final response errors (more than two standard deviations above the sample mean).

#### Materials

Three hundred recognizable silhouette images (e.g., animals, objects, figures) were obtained from a royalty-free clip art website (https://clipart-library.com/) and the stimuli used by a previous study (Oberauer et al., 2017). All the images were rescaled to 250 pixels × 250 pixels and set to white on the black background. A 360° color wheel was generated by the computer with the three primary colors (i.e., red, green, and blue) and their continuous mixtures using the hue, saturation and value (HSV) color model. At the beginning of the experiment, two hundred and eighty images were randomly selected from the image pool, with each of them being randomly painted with one of the colors on the color wheel for each participant. The 280 images were then randomly divided into two equal subsets with 140 images each. Within each subset, half images (i.e., 70) were randomly assigned to each condition (Test vs. Restudy).

#### Procedure

The experiment was implemented using Psychtoolbox in MATLAB 2022a (Kleiner et al., 2007). The experiment consisted of two identical runs, each involving participants studying 140 distinct images. Both runs followed the same three-phase procedure: Study, Practice (which involved either Test or Restudy), and Final Test. During the general instructions, participants were informed that feedback would be provided during practice and could help them evaluate their memory. Prior to the formal experiment, participants completed a practice session to familiarize themselves with the procedure.

During the Study phase, participants were instructed to memorize the colors of images, with each image presented once for 2 s. After every 10 images, participants were informed whether they would be tested on or restudy the preceding set. Half of all images (70 total, in seven blocks) were randomly assigned to Test and half to Restudy, for a total of 14 blocks. The order of Test and Restudy blocks was randomized by computer and unique to each participant.

In Test blocks, images reappeared in white (with colors removed) at the center of the screen, surrounded by a randomly rotated color wheel. As participants moved the mouse cursor along the color wheel, the centered image would be updated to preview that color selection. Participants provided their final decisions with a single, irreversible mouse click, with no time limit but an emphasis on accuracy. Immediate feedback followed for 1.5 s, showing the correct answer on the left side of the screen and their chosen color on the right side of the screen. This pairwise feedback format was used because the critical feedback information was the continuous color discrepancy between the selected and correct colors, which could be difficult to evaluate accurately if participants had to maintain their own response in working memory.

Restudy blocks followed a similar procedure, with one exception: Images were shown in their studied colors instead of white. Participants simply needed to locate and click the matching color on the wheel rather than recall it.

After finishing the test or restudy of all ten images in a block, participants began learning the images in the next block until all 14 blocks were finished.

After completing the study and subsequent test or restudy tasks for all 14 blocks of images, participants took a 5-min break before the final test. During the Final Test, all studied images appeared in white in a random order. Participants needed to restore each image’s original color, following the same procedure as the Test phase but without feedback.

**Fig. 2:**
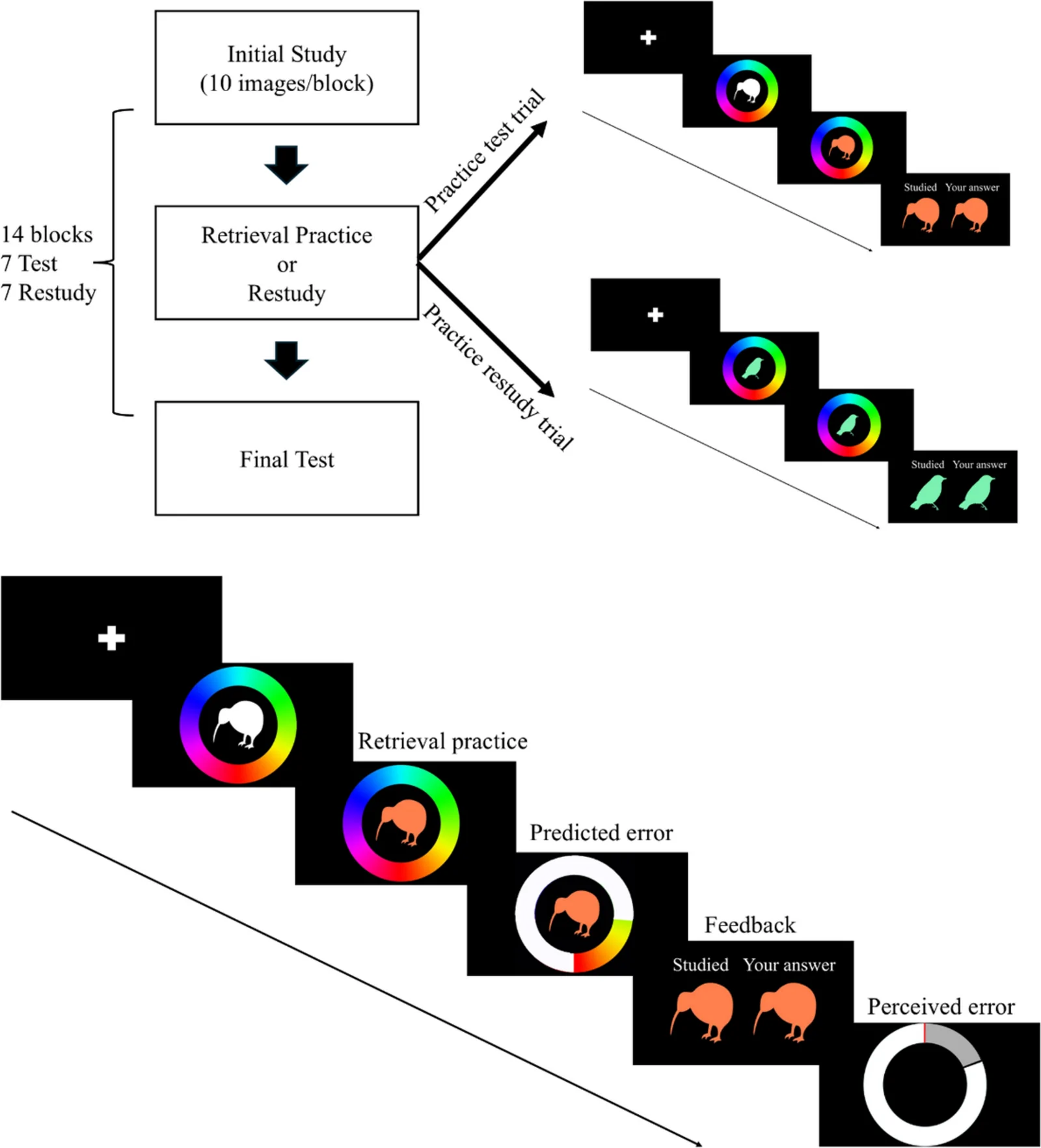
Experimental procedure. The upper panel shows the procedure of Experiment 1. The lower panel illustrates the procedure of a single practice test trial in Experiment 2. (Color figure online)

The second run began immediately after the conclusion of the first run. The entire experiment, comprising both runs, took approximately 1.5 h to complete (Fig. 2).

#### Data analyses

**Fig. 3:**
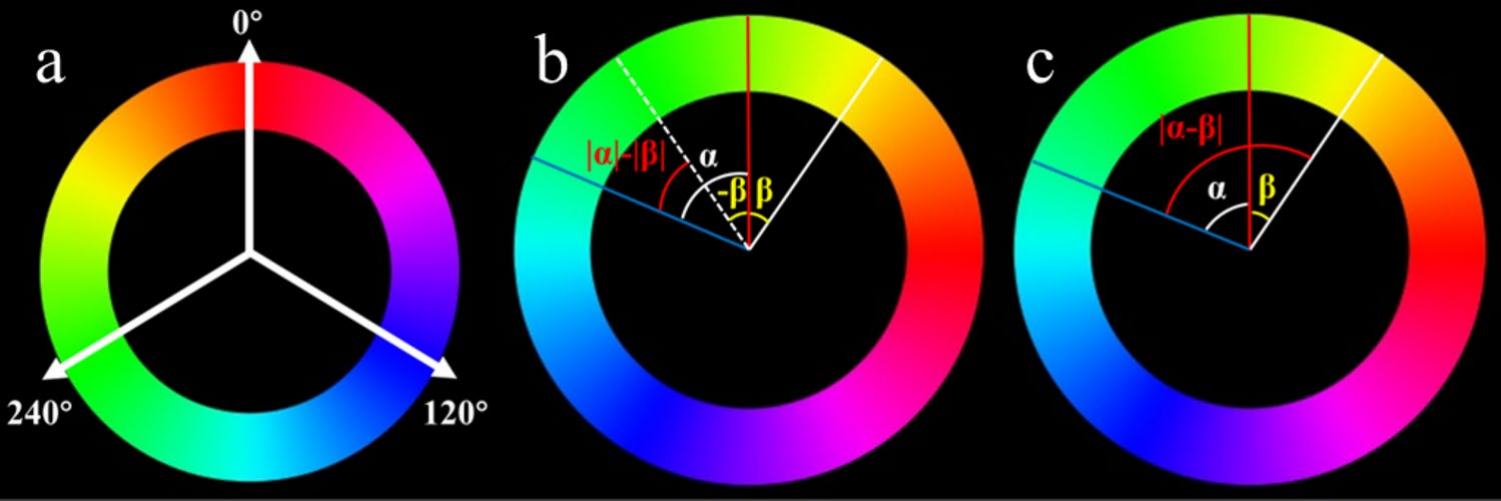
Angular distances measurement. **a** Colors are mapped to angular values on a 360° color wheel, with red set to 0°, increasing clockwise. Standard blue and green correspond to 120° and 240°, respectively, allowing each color to be uniquely represented by an angle. **b** Illustration of error reduction from practice to final test. The red line represents the target color, the solid blue line indicates the initial (practice) response, the white line represents the final response, and the dashed line is its mirror. α is the practice test error, and β is the final test error, both ranging from − 180° to 180°, with negative values indicating counterclockwise deviations. The quantity |α| – |β| captures the change in accuracy. **c** Angular distance between the practice and final responses, serving as another index of memory updating. Larger angular distances reflect greater change, which can also predict shifts in memory quality. (Color figure online)

The color wheel paradigm, in which each color corresponds to a unique angle on the color wheel, allows for converting recalled colors into continuous numerical values. For instance, if we align 0° to the standard red and measure clockwise, standard blue corresponds to 120° and standard green to 240° (see Fig. 3 for a detailed illustration).

Memory performance is quantified by calculating the absolute angular difference (ranging from 0 to 180°) between the studied color and the participant’s recalled color, termed the response error. For example, if a studied color is standard red and a participant selects either pink or yellow (both approximately 30° from red, but in opposite directions), the response error would be 30° regardless of direction. Lower response errors indicate better memory performance, allowing for a direct comparison of memory strength between Test and Restudy conditions. To examine changes from practice test to final test, we used two complementary measures.

1. Response error difference (practice test vs. final test): The first method calculates the difference between response errors in the Practice and Final Test phases (Fig. 3b). This measure directly indexes improvement in accuracy: Positive values indicate that the final response was closer to the target than the practice response. However, this measure is constrained by the initial practice error, because items with larger initial errors have more room to improve, creating a potential regression-to-the-mean concern.
2. Angular distance between practice and final test responses (Fig. 3c): Unlike response error difference, this measure does not indicate whether the final response became more accurate. Instead, it captures the magnitude of memory updating, that is, how much the remembered color shifted from practice to final test. This distinction is important for the EDL account, because the central prediction is that larger error signals should drive larger updates to the memory representation. These updates may produce improved accuracy, but they may also reflect partial correction, overcorrection, or other shifts in memory. Thus, symmetric final errors such as − 20° and + 20° indicate equivalent final accuracy, but they may reflect different updating trajectories when considered relative to the initial practice response.

Our analysis first examined the testing effect by comparing final response errors between restudied and tested items. We then examined whether practice-phase response errors predicted subsequent memory change using both response error difference and angular distance. Because response error difference is more vulnerable to initial error constraints, whereas angular distance more directly captures memory updating, we report both measures but place greater emphasis on angular distance for testing the EDL prediction. To further address the possibility that the observed effects reflect changes in final memory precision rather than updating magnitude, we also conducted an analysis of signed final-test errors by applying a von Mises distribution, which is reported in the Supplementary Materials.

### Results

#### The testing effect

**Fig. 4:**
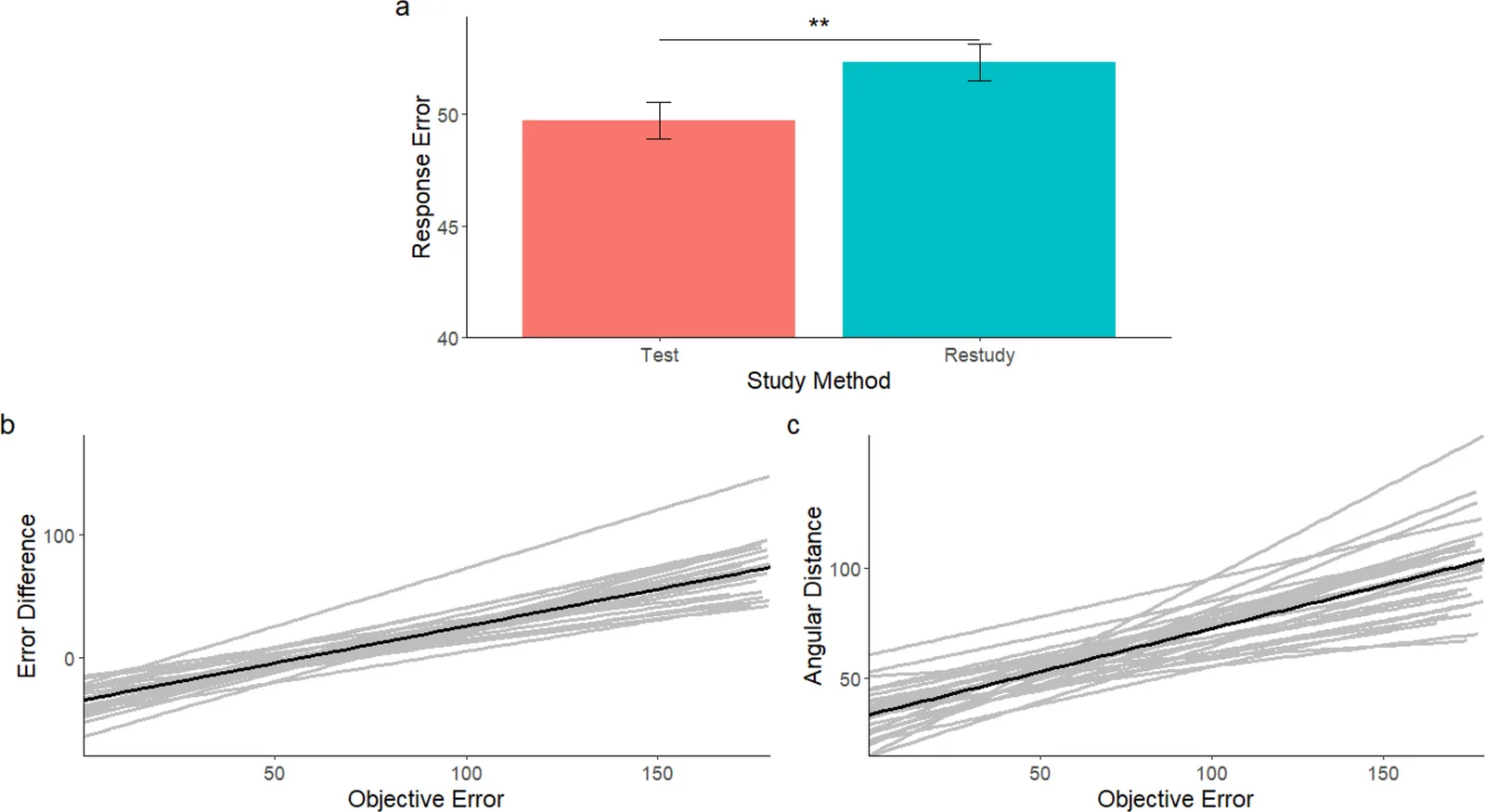
Results of Experiment 1**. a** The difference in the final recall performance between Test and Restudy conditions. Error bars represent standard errors. **p =.005. **b** The relationship between OE and the reduction in error from practice to final test (|α| – |β|). The black line shows the group-level trend; grey lines show individual participants. **c** The relationship between the OE and the angular distances. As in b, the black line reflects the group-level trend, with individual participants shown in grey. (Color figure online)

A paired-sample *t* test was conducted to compare memory performance in the final test between the Test and Restudy conditions. The analysis showed significantly lower average response errors for tested items (*M*_error_ = 49.71, *SD*_error_ = 12.21) compared with the restudied items (*M*_error_ = 52.32, *SD*_error_ = 13.49), *t*(28) = 3.07, 95% CI [.87, 4.35], *p* =.005, Cohen’s *d* = 0.57, BF_10_ = 8.72 (Fig. 4a). This replication of the testing effect set the stage for further examination of the EDL hypothesis.

#### The error-driven learning hypothesis

The EDL hypothesis predicts that larger response errors during practice should lead to greater changes in memory performance during the final test. Because response errors in the Restudy condition involved only perceptual matching rather than memory recall, our analyses focused solely on items in the Test condition.

We fitted linear mixed-effects models with participant identity as the random intercept. The first model examined how practice test errors predicted the difference between practice test and final test errors. The results showed a positive relationship (*β* =.6234, *t* = 36.90, *p* <.001; Fig. 4b). The second model, using angular difference as the dependent variable, similarly revealed that the practice test errors positively predicted response changes (*β* =.3718, *t* = 21.72, *p* <.001; Fig. 4c).

These findings indicate that errors can effectively enhance learning, with larger errors leading to greater memory updates. This supports the positive relationship between OE and final memory performance, as proposed by the EDL hypothesis (Table 1).

**Table 1:** Regression coefficients of models in Experiment 1

Model	Fixed effects	*β*	*t*	*p*	Variance
1	(Intercept)	− 35.22	20.80	<.001	54.73
	OE	.6234	36.90	<.001	2265.22
2	(Intercept)	34.18	19.35	<.001	61.35
	OE	.3718	21.72	<.001	2321.79

Model 1: the outcome variable is error difference. Model 2: the outcome variable is angular distance. Random effect: Participant

## Experiment 2

### Method

Experiment 1 provided preliminary evidence that the practice test errors enhance later recall performance. However, it remains unclear whether the OE we measured in Experiment 1 fully captured all the error signals that learners actually utilized. As discussed in the Introduction, a learner’s subjective expectation of their errors, the predicted error (PredE), can also serve as a potent driver of memory updating. For example, even if a learner’s actual retrieval error is only 5°, they may believe the error to be 45° due to low confidence or retrieval difficulty. In such cases, PredE can motivate learners to more strongly reject their initial response and seek other possibilities, thus leading to greater memory change than would be expected from the OE alone.

Additionally, we explored how subjective interpretation of feedback influences the utilization of errors, forming a more evidence-based version of subjective errors—Perceived Errors (PercE). Collectively, Experiment 2 examines the unique effects of OE, PredE, and PercE on memory performance, along with their interactions.

#### Participants

Forty participants were recruited for the formal experiment (28 women, *M*_age_ = 20.71 years). All participants reported normal or corrected-to-normal vision and normal color vision, provided informed consent before the experiment, and were compensated upon completion. A power analysis based on the testing effect comparison observed in Experiment 1 indicated that at least 35 participants were required to detect the effect with α =.05 and 95% power. To ensure consistency with Experiment 1 and increase statistical power for the trial-level mixed-effects analyses, we recruited 40 participants. We also conducted a sensitivity analysis for the mixed-effects design, which indicated that the current sample size and trial-level structure provided sufficient sensitivity to detect fixed effects of Cohen’s *d* = 0.45 or larger, suggesting adequate sensitivity for the trial-level mixed-effects analyses (Murayama et al., 2022).

#### Materials

The image pool was the same as in Experiment 1. However, in this experiment, participants studied 200 randomly selected images instead of 280 for difficulty and duration control purposes. Each image was randomly assigned to a unique color from the 360° color wheel, ensuring individualized color-image sets for each participant. The 200 images were then randomly divided into two equal subsets for testing.

#### Procedure

Similar to Experiment 1, participants followed the same Study-Practice Test-Final Test procedure in Experiment 2, but with key modifications. One major difference was the removal of the restudy condition, as subjective error measures (PredE and PercE) are only relevant in retrieval-based learning rather than passive restudy. Additionally, measures of PredE and PercE were introduced during the Practice Test phase to examine the role of subjective error cognition in learning.

After selecting a color for the given image, instead of receiving immediate feedback, they reported their predicted error (PredE) by eliminating colors they believed to be incorrect. Specifically, after retrieval, the image remained on the screen in the selected color, and participants clicked on colors they thought were incorrect, progressively removing them. Each click created a white line, and the regions between two white lines were replaced with white space, indicating elimination. The remaining uneliminated colors reflected their subjectively perceived correct range—the smaller this range, the greater their confidence in their chosen color. The entire elimination procedure was self-paced, and participants were allowed to eliminate as many regions as they wanted until only the colors they believed to have a chance to be correct remained.

After finalizing the PredE phase, participants received feedback as in Experiment 1. Before the task, participants had been informed that they would later report their perceived error; thus, the PercE judgment was intended to capture the subjective discrepancy formed during the feedback display. They then estimated their PercE by indicating how far their response deviated from the correct color. Participants were shown a white wheel, with a bold red line at the top center, representing the correct color’s location (0° error). They clicked on the wheel to report the perceived angular distance between the correct answer and their initial response. After clicking, the region between the red line and their selected position turned gray, visually illustrating the perceived error. A smaller gray region indicated a lower perceived error, whereas a larger gray region indicated a greater perceived discrepancy between their response and the correct answer.

After completing all Study and Practice blocks, participants performed a 10-min working memory capacity (WMC) task before proceeding to the final test, serving as the distractor task.

#### Data analyses

Similar to Experiment 1, we calculated absolute response error differences and angular distance to describe the memory performance. In addition to OE, in Experiment 2, we also incorporated two additional subjective error measures: PredE and PercE. Specifically, OE was defined as the response errors during the practice test, PredE represented the sum of the remaining uneliminated regions in the color-elimination task, and PercE reflected the self-reported discrepancy between the retrieved and correct colors after feedback. Hence, the ranges for OE and PercE are [0,180] while the range for PredE is [0,360].

To examine the relationship between these error types and memory performance, we first replicated the regression analysis from Experiment 1, assessing whether OE continued to predict memory performance. Next, we added PredE and PercE alongside OE to test their independent contributions to memory strengthening during and after the practice test. Finally, we analyzed the interactions among all three types of errors to explore how these factors influenced learning outcomes.

### Results

**Fig. 5:**
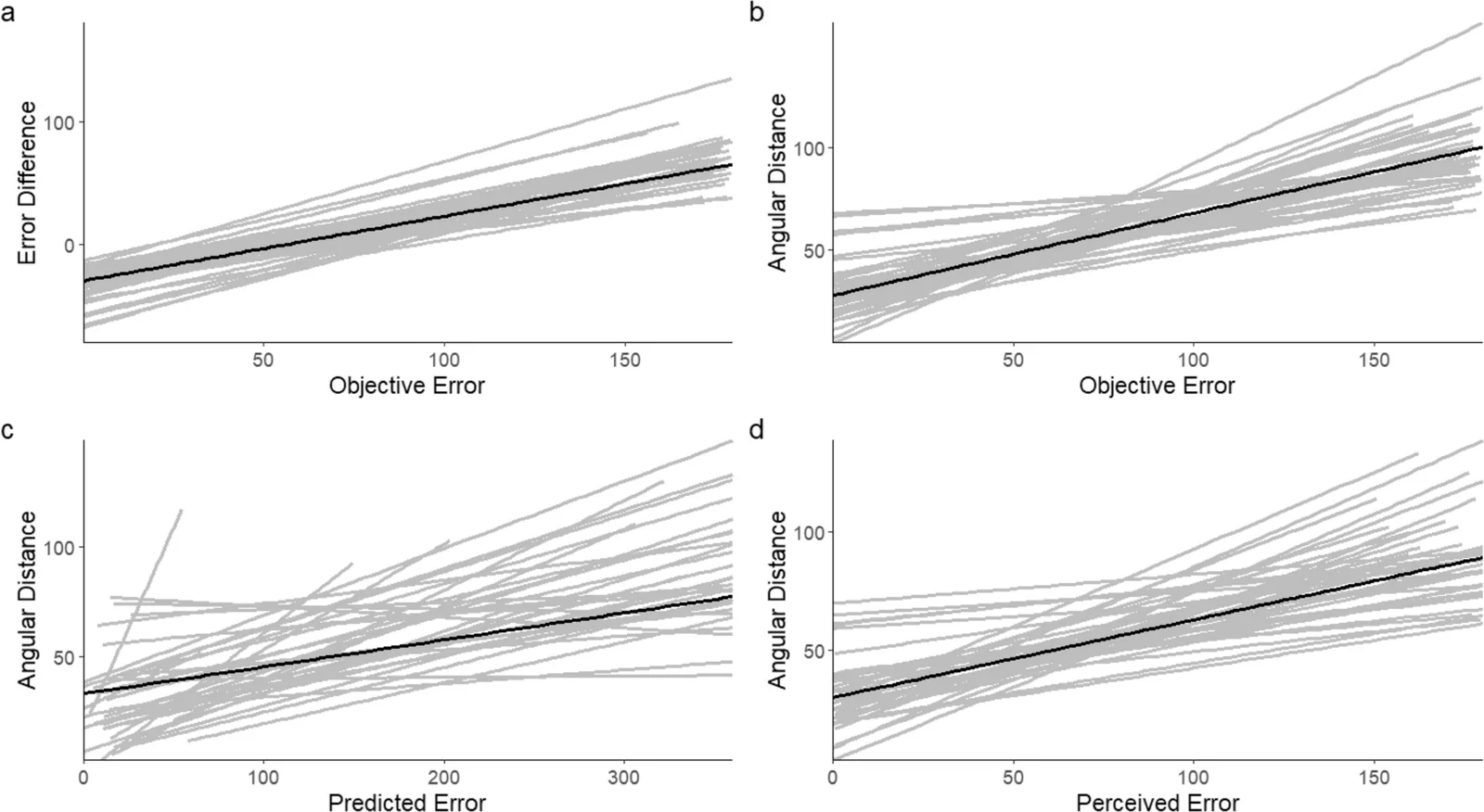
Results of Experiment 2. **a** The relationship between OE and the reduction in error from practice to the final test. **b** The relationship between OE and the angular distance between the practice and the final test responses. **c** The relationship between PredE and angular distance. **d** The relationship between PercE and angular distance. In all panels, the black line represents the group-level trend, and the grey lines represent individual participants

To replicate the findings of Experiment 1, we constructed two linear mixed-effects models, with participant ID as the random intercept. The first model examined the relationship between OE and error differences, revealing a significant positive association (*β* =.5793, *t* = 51.06, *p* <.001), consistent with the results from Experiment 1 (Fig. 5a). The second model replaced error differences with angular differences, which similarly showed a significant positive effect (*β* =.3576, *t* = 30.66, *p* <.001). These results reinforce the notion that larger practice errors predict greater updating in memory performance.

Next, we included PredE and PercE as predictors in the linear mixed-effects models to examine whether the subjective errors uniquely predicted memory updating after controlling for the others. The main effect model showed significant main effects for all three predictors (*β*_OE_ =.2583, *t* = 13.38, *p* <.001; *β*_PredE_ =.0934, *t* = 10.61, *p* <.001; *β*_PercE_ =.0787, *t* = 4.313, *p* <.001; Fig. 5b–d), suggesting both PredE and PercE can positively predict memory updating even after controlling for OE. Notably, variance inflation factors (VIFs) for these predictors were 2.78 (OE), 1.07 (PredE), and 2.78 (PercE), indicating an acceptable level of multicollinearity.

**Fig. 6:**
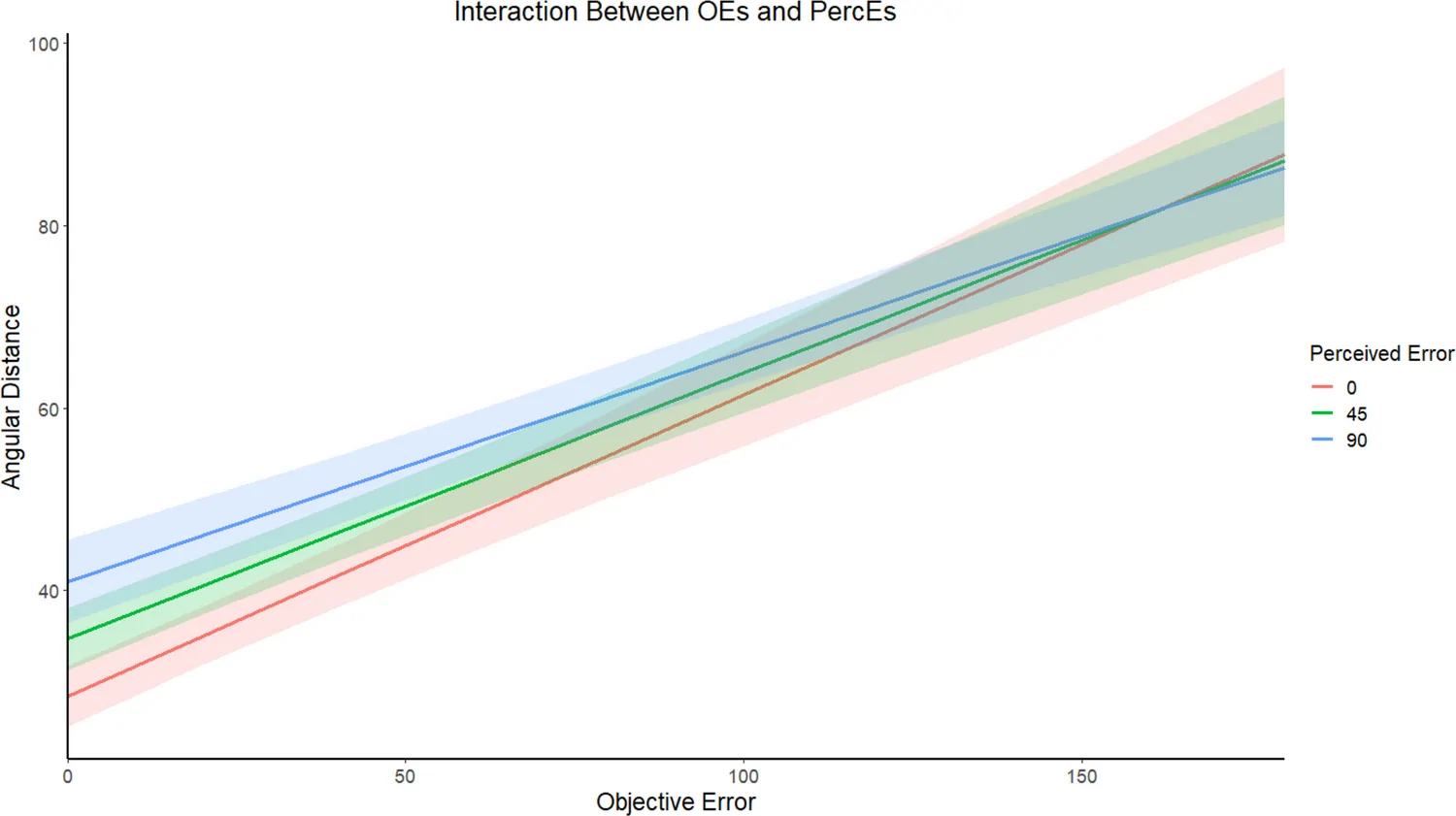
Interaction between OE and PercE in predicting angular distance. (Color figure online)

Further, we tested interaction effects among OE, PredE, and PercE using a three-way interaction model. This model did not reveal a significant three-way interaction (*β* =.000004, *p* =.151). We then examined two-way interactions by fitting a model that included only main effects and two-way interactions. The analysis revealed a significant interaction effect between OE and PercE (*β* =  −.0009, *p* <.001; Fig. 6). Additionally, the interaction between PredE and PercE was marginally significant (*β* =  −.0004, *p* =.050). A simple slope analysis of the interaction indicated that the effect of PercE on memory performance was larger when OE was small but diminished as OE increased (see Table 2 for regression coefficients of all regression models in Experiment 2). To examine the robustness of these interaction effects, we reanalyzed the models using the Kenward–Roger approximation for degrees of freedom, a more conservative method for assessing significance in linear mixed-effects models (Luke, 2017). The conclusions remained largely unchanged; these analyses are reported in the Supplementary Materials (Table S1).

**Table 2:** Regression coefficients of the main effect models (Model 1, 2, and 3), the two-way interactions model (Model 4), and the three-way interaction model (Model 5) in Experiment 2

Model	Fixed effects	*β*	*t*	*p*	Variance
1	(Intercept)	− 31.75	18.81	<.001	95.61
	OE	.5793	51.06	<.001	2,047.56
2	(Intercept)	29.71	18.88	<.001	79.5
	OE	.3576	30.66	<.001	2,168.9
3	(Intercept)	22.22	12.29	<.001	94.7
	OE	.2583	13.38	<.001	2,130.6
	PredE	.0934	10.61	<.001	
	PercE	.0787	4.31	<.001	
4	(Intercept)	17.83	9.48	<.001	82.0
	OE	.3444	9.51	<.001	2,124.0
	PredE	.1227	10.22	<.001	
	PercE	.1734	5.70	<.001	
	OE*PredE	−.0002	− 0.78	.433	
	OE × PercE	−.0009	− 3.40	<.001	
	PredE × PercE	−.0004	− 1.96	.050	
5	(Intercept)	16.87	8.47	<.001	81.2
	OE	.3793	8.71	<.001	2,123.8
	PredE	.1341	9.30	<.001	
	PercE	.1991	5.64	<.001	
	OE × PredE	−.0005	− 1.58	.113	
	OE × PercE	−.0013	− 3.31	<.001	
	PredE × PercE	−.0007	− 2.41	.016	
	OE × PredE × PercE	.000004	1.44	.151	

Model 1: the outcome variable is error differences. Others: the outcome variable is angular distances. Random effect: Participant

## Model simulation

### Method

We adapted the dual-process model of the testing effect recently proposed by Zheng et al. (2023, 2024) to simulate the learning process. This model, known as the Working memory-dependent Retrieval Attempt and Postretrieval processing (WRAP) model, describes the two processes of test-enhanced learning: retrieval attempt and postretrieval reencoding. These stages align with the generation of PredE and PercE, respectively, in the current study. Given that our model is an extension of the original WRAP model, incorporating the role of error-driven learning, we refer to it as the WRAP-E(error) model.

**Fig. 7:**
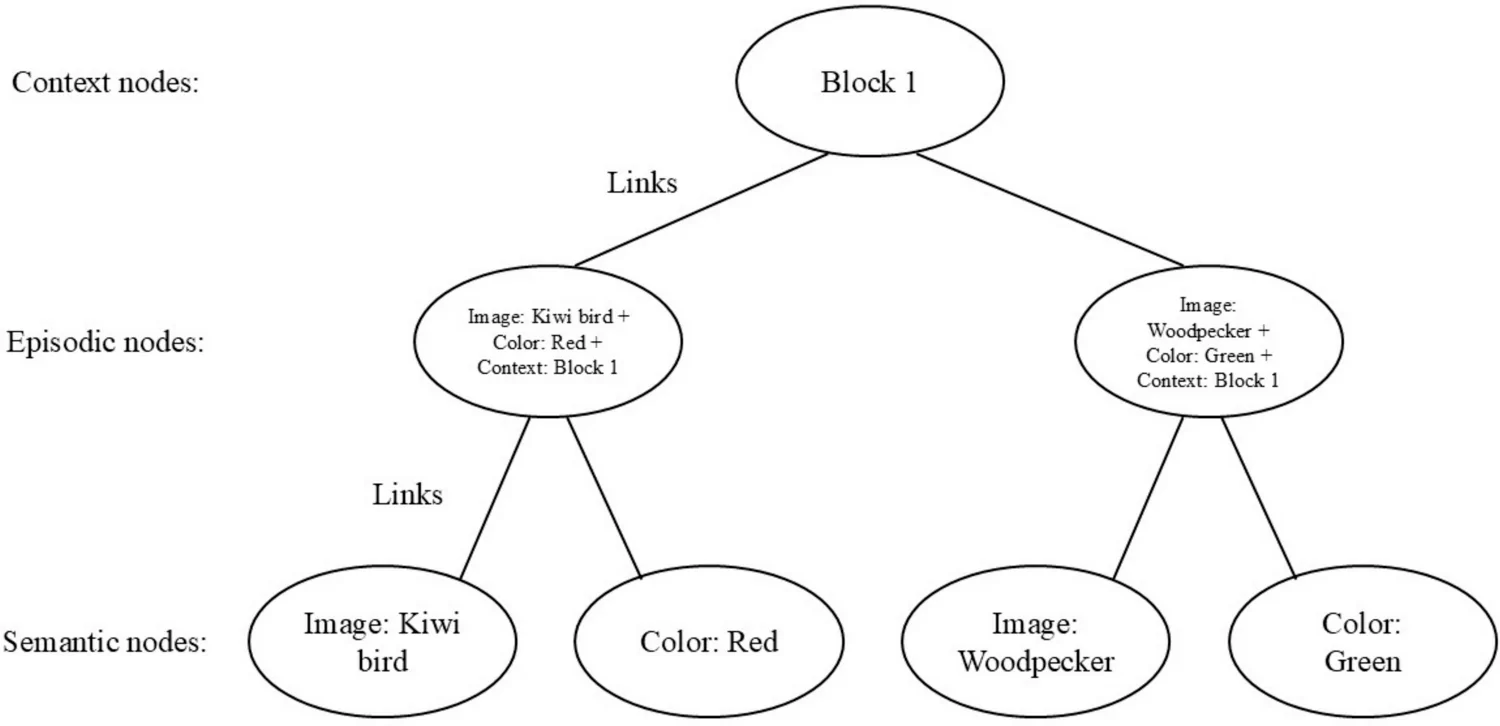
Illustration of the WRAP-E model structure**.** During the study phase, participants learned color–image associations (e.g., red–kiwi bird, green–woodpecker). Each image and color are represented as a semantic node, which links to an episodic node encoding the specific color–image pair. These episodic nodes are, in turn, bound to a context node representing the study block (Block 1). Notably, while each image appears in only one episode, the same color may be linked to multiple episodic nodes, reflecting its reuse across different trials. This structure captures the hierarchical relationships among context, episode, and semantic information in memory encoding

The core idea of the WRAP model is inherited from the Source of Activation Confusion (SAC) model (Popov & Reder, 2020), which conceptualizes episodic memory as a localist network consisting of interconnected conceptual, episodic, and contextual nodes (Fig. 7). The conceptual nodes serve as the basis of the network, with multiple conceptual nodes connecting to distinct episodic nodes, which, in turn, connect to a shared contextual node when they occur within the same context. For instance, in our experiment, unique images and colors are represented as conceptual nodes, while color-image pairs are represented as episodic nodes, and the study blocks are represented as contextual nodes. Each node has a base-level strength, representing its storage strength, and an activation strength, which determines its retrieval probability. When relevant information is processed, the corresponding nodes are activated, and activation spreads connected nodes, reinforcing both their base-level and activation strengths. For example, if a participant studies a red kiwi bird in the study phase, the corresponding nodes for kiwi bird" (conceptual node), "red-kiwi bird" (episodic node), and the study block context (contextual node) are created. Later, during the test phase, when the participant encounters an uncolored kiwi bird, the kiwi bird conceptual node and the red-kiwi bird episodic node are activated, strengthening their associations. The activation then spreads to other connected nodes, such as the red color conceptual node and the contextual block node.

A key feature of the WRAP model is that memory strengthening occurs twice, once during the retrieval attempt process and once during the postretrieval reencoding. Moreover, the strengths decay over time, simulating forgetting. Similar to nodes, the links between nodes also have strengths determined by how frequently the connected nodes are co-activated, and the link strengths will decay over time as well. The activation of nodes can spread to other connected nodes via the links between them, and the spreading activation strengths are the product of the link strengths and the activation strengths (see Popov & Reder, 2020; Zheng et al., 2023, 2024, for details).

To adapt the WRAP model for error-driven learning, we introduced several modifications. First, in the original WRAP model, the memory performance was treated as binary (correct/incorrect) and determined by memory strengths constrained between 0 and 1. In the present study, however, memory performance was measured continuously using absolute angular error. Accordingly, we reformulated the model’s memory-state variable in units of error rather than conventional memory strength: decreases in absolute error represented memory improvement, whereas increases represented memory deterioration. Moreover, since our focus was on learning through practice tests rather than initial encoding, we simulated the learning process only after the initial study phase. The initial base-level strengths were set as the observed retrieval practice errors:

1 $${B}_{0}=OE.$$

Here,* B*_0_ is the initial base-level strength after the initial study, and *OE* is the observed errors in practice tests (i.e., objective errors). This modification replaced the estimated episodic node strengths in the original model with directly observed errors, allowing for trial-by-trial simulation of learning processes.

Second, because we are primarily interested in the relationship between errors and angular distances between practice test and final test responses, our focus shifted from estimating retrieval probability (in the original WRAP model) to predicting changes in angular distance between practice and final test responses. This outcome was captured through Eqs. 1 and 2. Memory strengthening in the current model was represented by a reduction from the original error toward zero at a certain learning rate *φ*:

2 $$s= -{B}_{0}\times \varphi .$$

The angular distance was calculated as the sum of all strengthening increments adjusted for temporal decay:

3 $$A=\sum\nolimits_{i=1}^{n}|s|\times {(1+t-{t}_{i})}^{-d},$$

where *d* is the decay rate, *t* is the onset time of the current trial, and *t*_*i*_ is the onset of the event when the *i*-th strengthening took place. This strengthening process can also naturally capture overcorrection: Because the response is updated toward the correct value, a sufficiently large update may move it past the correct value and to the opposite side. In the original WRAP model, the temporal decay rate was set to 0.18, appropriate for a memory strength scale ranging from 0 to 1. However, in the current model, angular distances range from 0 to 180 degrees. This expanded scale made the original parameter incompatible with the model, resulting in implausible estimates of other parameters. To address this, a new decay rate was estimated.

Third, according to the EDL hypothesis, subjective errors influence memory updating jointly with OE. Specifically, PredE reflects the learner’s subjective expectation of error magnitude before feedback. It may modulate the impact of OE on memory updating, such that stronger PredE amplifies the effect of OE when feedback is provided. Similarly, PercE reflects the learner’s subjective interpretation of the feedback and may further guide memory updating during the subsequent post-feedback phase.

To formalize this, we modeled the memory updating increment using the following equations. The first stage spans from the initiation of the retrieval attempt until the presentation of feedback. During this stage, the memory updating increment is computed as the OE scaled by its associated learning rate *φ*_OE_, augmented by PredE scaled by *φ*_PredE_ (Eq. 4):

4 $$s=-\left(OE\times {\varphi}_{OE}+PredE\times {\varphi}_{PredE}\right).$$

The second stage is postretrieval reencoding after feedback is provided. PercE takes the place of OE for further updating. The increment is thus modeled simply as PercE scaled by its corresponding learning rate *φ*_PercE_.

5 $$s=-PercE\times {\varphi}_{PercE}.$$

Consistent with the original WRAP model, each node was strengthened twice, reflecting the retrieval attempt and postretrieval reencoding processes. The final angular distance was then computed by summing both decayed increments. To evaluate whether this stage assignment was necessary, we also fitted an alternative model in which error components were assigned to different stages (Table S2).

The fourth modification concerned the working memory capacity parameter. In the original WRAP model, this parameter limits the amount of information that can be actively maintained and used during both stages. In the present WRAP-E model, it serves a related scaling function by constraining the maximum gain applied to error-driven memory updates. Because the current paradigm represents memory strength on an angular-error scale ranging from 0° to 180°, the original value used in WRAP produced implausibly small strengthening increments. We therefore rescaled this parameter to fit the present error scale and treated it as constant across participants, because individual differences in working memory capacity were not the primary focus of the study. To ensure that this choice did not drive the main conclusions, we also fitted an alternative model in which this parameter was allowed to vary across participants; this model is reported in the Supplementary Materials (Table S2). Other aspects of the model remain the same as the original WRAP model (Popov & Reder, 2020; Zheng et al., 2023, 2024).

### Model fitting and testing

All model parameters (i.e., *φ*s for OE, PredE, and PercE and the decay rate *d*) were first estimated at the individual level. We iterated through all plausible values of *φ*s (range from 0 to 1 with a step size of 0.05) and *d* (range from 0 to 0.1 with a step size of 0.001). The optimal individual parameter set was selected by minimizing the root-mean-square error (RMSE) between the simulated and observed angular distances. After obtaining individual estimations, we averaged the four parameters across participants to determine the final parameter set and used this set to reestimate the angular distances across all trials for all participants (Table 3).

The model was evaluated by comparing simulated results to empirical data. We replicated the Experiment 2 analyses using the simulated angular distances to examine how well the WRAP-E model reflected the real learning processes during testing.

Although our primary focus was memory updating, indexed by angular distance, we also examined whether the model could reproduce final response errors in the Test condition. To do so, we derived each final response from the corresponding practice test response and the fitted angular distance. The resulting final error values were comparable to the observed data, as reported in the Supplementary Materials (Table S3).

The current model, however, cannot be directly applied to the Restudy condition. Because restudy does not require active retrieval, no trial-level objective or subjective retrieval errors are available during practice. As these error signals are the key inputs to WRAP-E, the model cannot directly predict final memory performance in restudy or fully simulate the testing-versus-restudy contrast observed in Experiment 1.

**Table 3:** Fitted parameters for the WRAP-E model

*φ*_OE_	*φ*_PredE_	*φ*_PercE_	*d*
0.33	0.62	0.52	0.05

### Results

**Fig. 8:**
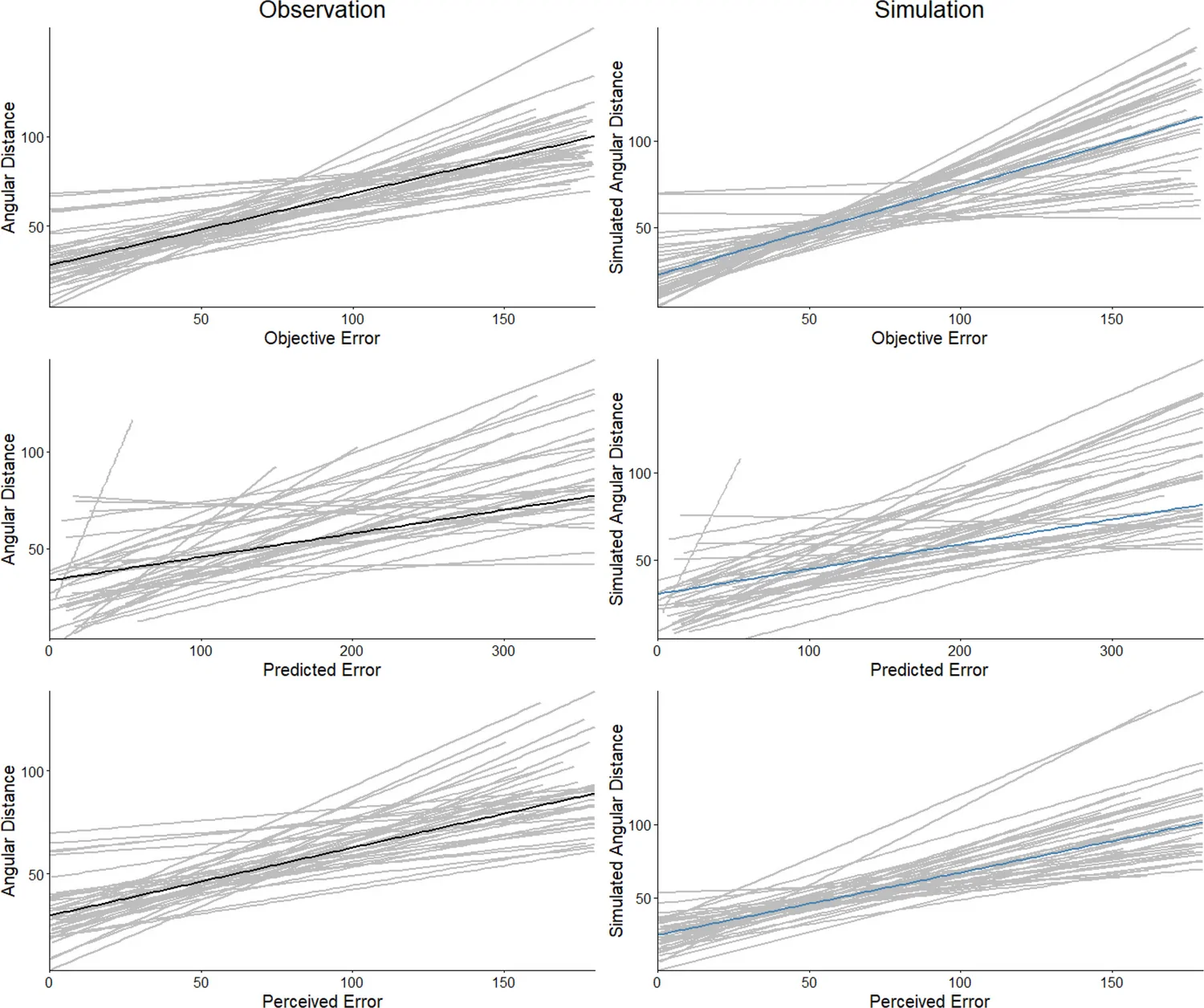
Observed vs. simulated relationships between error metrics and angular distance. Left panels show the empirical data, and right panels show the corresponding model simulations generated using the WRAP-E model. Each row displays the relationship between (simulated) angular distance and a different error type

Regression analyses on the simulated data, as shown in Fig. 8 and Table 4, replicated key empirical patterns. In the main effect model, consistent with the empirical data, all three predictors (OE, PredE, and PercE) showed significant effects (*β*_OE_ =.5851, *t* = 47.74, *p* <.001; *β*_PredE_ =.0447, *t* = 7.98, *p* <.001; *β*_PercE_ =.0387, *t* = 3.34, *p* <.001). However, different from the empirical data, the three-way interaction model showed a significant three-way interaction but in the same direction as observed (*β* =.00002, *t* = 8.31, *p* <.001).

**Table 4:** Regression coefficients of the main effect models (Model 1 and 2), the two-way interactions model (Model 3), and the three-way interaction model (Model 4) fitted with predicted angular distances from the WRAP-E model

Model	Fixed effects	*β*	*t*	*p*	Variance
1	(Intercept)	23.75	20.74	<.001	43.37
	OE	.4821	60.49	<.001	1,011.27
2	(Intercept)	1.538	12.58	<.001	50.57
	OE	.3313	25.51	<.001	963.48
	PredE	.0896	15.09	<.001	
	PercE	.1420	11.55	<.001	
3	(Intercept)	5.396	4.43	<.001	34.05
	OE	.6076	25.66	<.001	907.61
	PredE	.1359	17.32	<.001	
	PercE	.3900	19.60	<.001	
	OE × PredE	−.0004	− 2.70	.007	
	OE × PercE	−.0030	− 17.88	<.001	
	PredE × PercE	−.0006	− 4.62	<.001	
4	(Intercept)	1.770	1.38	.171	33.13
	OE	.7387	26.05	<.001	900.02
	PredE	.1789	19.08	<.001	
	PercE	.4865	21.19	<.001	
	OE × PredE	−.0016	− 7.92	<.001	
	OE × PercE	−.0046	− 18.01	<.001	
	PredE × PercE	−.0017	− 9.14	<.001	
	OE × PredE × PercE	.00002	8.31	<.001	

These simulated interactions clarify the mechanism captured by WRAP-E. The interactions were not imposed as regression terms, but emerged from the model’s sequential and bounded updating process: once OE or PredE have already produced substantial updating, less discrepancy remains for PercE to further shift memory. This explains the negative interactions involving PercE, consistent with the empirical pattern. The simulated three-way interaction reached significance whereas the empirical one did not, likely because simulated data contained less trial-level noise. Thus, the model captures the broad structure of sequential error-driven updating rather than the exact statistical pattern of every interaction.

## Discussion

This study examined the error-driven learning (EDL) hypothesis of the testing effect through two experiments. Experiment 1 revealed a positive correlation between practice test errors and updates in the final recall. Experiment 2 further investigated the joint contributions of objective errors (OE), predicted errors (PredE), and perceived errors (PercE) to memory strengthening. Additionally, we quantitatively modeled these findings with the WRAP-E model.

### Retrieval practice error and memory performance

Extensive empirical evidence underscores the role of errors in enhancing memory performance. For instance, prior research has demonstrated that forced guessing prior to formal study improves learning outcomes, even when guesses are incorrect—a phenomenon termed the pretesting effect (Cyr & Anderson, 2012; Huelser & Metcalfe, 2012; Liu et al., 2021; Pan & Rivers, 2023; Richland et al., 2009). Furthermore, Cyr and Anderson (2015) showed that errors from guessing enhance retention in older adults, challenging earlier assumptions that errors impair memory in aging populations (Clare & Jones, 2008; Middleton & Schwartz, 2012). Bahrick and Hall (2005) found that the errors during retrieval practice correlate positively with the magnitudes of the spacing effect, where longer intervals between study sessions yield greater memory benefits (see also Antony et al., 2024). The benefits of error generation extend across modalities. For example, Herzfeld et al. (2014) demonstrated that errors drive sensorimotor learning. Szpunar et al. (2014) highlighted the benefits of erroneous recall in visual episodic memory.

Building on the empirical findings, Liu et al. (2021) proposed the EDL hypothesis of the testing effect, which posits that practice tests generate more errors than restudy, thereby enhancing memory performance. Experiment 1 of the current study provided direct support for this hypothesis, demonstrating that retrieval practice error magnitudes positively predict memory updates.

### The role of subjective errors in retrieval practice

OE does not fully capture the learning-relevant signals, as learners’ metacognitive judgments often diverge from actual performance (Barenberg & Dutke, 2019; Chen et al., 2019; Little & McDaniel, 2015; Rivers, 2021). Since learners’ subjective evaluations influence how information is processed and retained, a more rigorous test of the EDL hypothesis requires examining whether memory updating is shaped not only by OE but also by subjective errors, those informed by metacognitive monitoring.

In Experiment 2, we operationalized subjective errors as PredE and PercE, both rooted in metamemory—the self-assessment of memory processes and quality (Dunlosky et al., 2015; Jacoby et al., 2010; Little & McDaniel, 2015). These two forms of errors are distinguished by the timing and the informational basis of their formation. PredE arises from the interplay between OE and subjective experiences during tests. Once an answer is submitted, the OE is fixed, but learners infer its size based on confidence and fluency: high-confidence errors are often underestimated, while low-confidence responses lead to inflated error judgments. Prior research suggests that test-taking inherently prompts performance evaluation, making PredE an integral part of retrieval-based learning (Barenberg & Dutke, 2019; Miller & Geraci, 2014, 2016; Pan & Rivers, 2023).

PercE emerges after retrieval once learners receive the feedback and directly compare their recalled answers to the correct answers. This comparison yields a more accurate error assessment, correcting biases in PredE (Bahrick & Hall, 2005; McDonough et al., 2021; Tullis et al., 2013). However, our results indicate that PercE may still differ from OE, which suggests that even with explicit feedback, error perception remains influenced by metacognitive biases.

Therefore, subjective errors evolve dynamically throughout retrieval practice. Initially, retrieval attempts generate OE, serving as the foundation for memory adjustments. Immediately after retrieval, learners form PredE, which reflect their metacognitive estimations of error magnitude and influence learning until feedback is provided. Once feedback is provided, the influence of PredE manifests immediately with the actual OE revealed by it. Then, PercE replaces PredE, refining learners’ understanding of their errors and influencing the postretrieval reencoding process. This progression highlights the critical role of both predictive and perceptual error monitoring in shaping memory strengthening, where subjective evaluations interact with objective errors to drive learning. This temporal distinction between PredE and PercE is also consistent with cognitive neuroscience work distinguishing internally generated error-monitoring signals, such as the error-related negativity, from feedback-based error signals, such as the feedback-related negativity. This parallel suggests that subjective error monitoring may unfold across separable prefeedback and postfeedback stages (Gehring et al., 1993; Holroyd & Coles, 2002; Holroyd et al., 2008; Miltner et al., 1997).

Building on this hypothesis, Experiment 2 empirically tested the distinct contributions of OE, PredE, and PercE to memory updating using mixed-effects linear models. In addition to replicating the role of OE observed in Experiment 1, the results revealed that PredE and PercE also significantly contributed to memory gains.

In addition to the main effects, we found interactions between PercE and both OE and PredE, indicating that PercE contributed more strongly to memory strengthening when OE or PredE was small. This pattern suggests that PercE plays a secondary role in learning: when objective or predicted errors have already driven substantial updating, there may be less remaining room for perceived errors to further strengthen memory. This interpretation aligns with the dual-process account proposed in the original WRAP model, in which PercEs primarily contribute during postretrieval reencoding.

It should be noted, however, that these interaction effects were small in magnitude, despite reaching statistical significance. We therefore interpret them cautiously. Rather than placing strong emphasis on the exact size of these effects, we view them as preliminary evidence that subjective error signals may modulate how other error signals contribute to memory updating.

The present findings also relate to the broader literature on prediction error and memory. Prediction error generally refers to a mismatch between what a learner expects and what is actually encountered, and prior work has shown that such mismatches can enhance learning, especially during encoding (e.g., Greve et al., 2017; Quent et al., 2021). In the present framework, prediction error can be conceptualized as the mismatch between expected and actual error, reflected by the discrepancy between PredE and OE (Shi et al., 2026). We did not observe a significant PredE × OE interaction, possibly because the arbitrary image–color associations used here provided relatively weak prior expectations. Stronger prediction-error effects may therefore emerge when learners have more robust prior knowledge or stronger confidence in their predictions.

One limitation of the current design, however, is that the metacognitive judgment procedures in Experiment 2 may have introduced reactivity (Double & Birney, 2019; Double et al., 2018). Estimating PredE and PercE may have increased participants’ attention to their responses and feedback, potentially strengthening error monitoring relative to standard retrieval-practice paradigms. Future work should test whether similar relationships among objective errors, subjective errors, and memory updating emerge with more implicit measures.

### The WRAP-E model for error-driven learning

To further examine error-driven learning, we developed a computational model, the WRAP-E model, which extends the original WRAP model to incorporate both objective and subjective errors. This model was fitted to the empirical data to quantitatively assess the mechanisms underlying the testing effect.

Simulations using the WRAP-E model reproduced the key patterns observed in Experiment 2, particularly the independent contributions of OE, PredE, and PercE to memory strengthening. These results suggest that subjective errors play a distinct role in retrieval-based learning beyond what can be captured by objective errors alone. Importantly, the model further suggests that interactions among error signals may arise from a sequential and bounded updating process, in which later error signals have less influence when earlier signals have already produced substantial memory change.

The WRAP-E model and the EDL hypothesis provide a mechanistic account of the testing effect that complements and may help reconcile inconsistencies across existing theoretical explanations. For example, the semantic elaboration account proposes that retrieval enhances memory by encouraging richer semantic processing (Carpenter, 2009; Pyc & Rawson, 2009), whereas episodic context accounts propose that retrieval benefits memory by reinstating and updating contextual representations (Karpicke et al., 2014; Lehman et al., 2014). These accounts explain many findings but are less directly applicable to cases in which retrieval benefits emerge for nonverbal materials with limited semantic structure, such as the image–color associations used here, or when focal target memory improves without a corresponding benefit in contextual memory (Gupta et al., 2022; Hong et al., 2019).

We view EDL as complementary to these accounts rather than mutually exclusive. Semantic elaboration and context reinstatement may influence what information is retrieved, compared, and updated, whereas error signals specify how discrepancies between the retrieved response and the correct target drive learning. Thus, WRAP-E provides a unified mechanistic account in which retrieval practice enhances memory because it generates informative error signals that guide adaptive updating, potentially alongside semantic and contextual processes.

In summary, this study provides support for the error-driven learning hypothesis through two behavioral experiments and a computational model. Both objective and subjective error magnitudes were found to predict improvements in memory performance, consistent with the idea that errors act as signals to drive adaptive memory updating. Although the WRAP-E model accounts for a wide range of findings, future research should further validate the causal role of error magnitude and refine the integration between cognitive and neural perspectives on error-based learning.

## Supplementary Information

Below is the link to the electronic supplementary material.

Supplementary file1 (DOCX 23 kb)

## Data Availability

The data and materials are publicly available online (https://osf.io/e352p).

## Code availability

The code is publicly available online (https://osf.io/e352p).
