# Supplementary material for: Learning from mistakes: Objective and subjective error magnitudes predict the testing effect
Source: Psychon Bull Rev. 2026 Sep 24;33(7):246. doi: 10.3758/s13423-026-03009-z (PMC13612663; doi:10.3758/s13423-026-03009-z)
Supplement: Supplementary file 1 [file 13423_2026_3009_MOESM1_ESM.docx]

**Supplementary Materials**

**Robustness Check Using the Kenward–Roger Approximation**

Because the interaction coefficients in Experiment 2 were small, we conducted an additional robustness check using the Kenward–Roger approximation for degrees of freedom, which provides a more conservative assessment of significance in linear mixed-effects models (Luke, 2017). As shown in Table S1, the results were substantively unchanged. The OE × PercE and PredE × PercE interactions remained significant, whereas the OE × PredE interaction and the three-way interaction remained non-significant.

**Table S1. Mixed-effects model with Kenward–Roger approximation**

| Fixed effects | *β* | *t*(*df*) | *p* | *t*(KR’s *df*) | KR’s *p* |
| --- | --- | --- | --- | --- | --- |
| (Intercept) | 16.87 | 8.47(106.0) | <.001 | 8.47(110.5) | <.001 |
| OE | .3793 | 8.71(7859) | <.001 | 8.70(7864) | <.001 |
| PredE | .1341 | 9.30(7051) | <.001 | 9.29(7086) | <.001 |
| PercE | .1991 | 5.64(7837) | <.001 | 5.64(7843) | <.001 |
| OE*PredE | -.0005 | -1.58(7982) | .113 | -1.58(7982) | .113 |
| OE*PercE | -.0013 | -3.31(7954) | <.001 | -3.30(7956) | <.001 |
| PredE*PercE | -.0007 | -2.41(7990) | .016 | -2.40(7990) | .016 |
| OE*PredE*PercE | .000004 | 1.44(7989) | .151 | 1.44(7989) | .151 |

*Note*. OE = Objective Error; PredE = Predicted Error; PercE = Perceived Error. KR = Kenward-Roger

**Alternative Models**

To evaluate whether the stage assignment of error signals was necessary for the WRAP-E model, we tested several alternative model specifications. As shown in Table S2, assigning error signals to different processing stages worsened model fit relative to the main model. Allowing the working memory capacity parameter to vary across individuals also did not improve fit after accounting for the additional model complexity, as reflected in higher AIC and BIC values. These results support the primary WRAP-E specification.

Table S2. Model Comparison

| Model | Description | RMSE | AIC | BIC |
| --- | --- | --- | --- | --- |
| 1 | OE + PredE at retrieval, PercE at feedback (Main model) | 27.60 | 75796.71 | 75831.65 |
| 2 | PercE at retrieval, OE + PredE at feedback | 32.53 | 78429.33 | 78464.26 |
| 3 | Model 1 with individual working memory capacity parameter | 27.71 | 75944.01 | 76258.44 |

*Note*. OE = Objective Error; PredE = Predicted Error; PercE = Perceived Error. Model 1 is the primary model reported in the main text. Lower RMSE, AIC, and BIC indicate better fit.

**Deriving Final Error from the WRAP-E Model**

Although the primary focus of the WRAP-E model was memory updating, indexed by angular distance, we also examined whether the model could reproduce final response errors. To do so, we first fitted angular distance and then derived the final response from the retrieval practice response.

The model assumes that updating begins from the retrieval practice response and proceeds toward the correct answer. Accordingly, the final response was computed by applying the cumulative strengthening increments, adjusted for temporal decay, to the retrieval practice response. In some trials, the cumulative update exceeded the initial retrieval practice error, producing an overcorrection beyond the correct answer. These cases were retained as overcorrections, and final response error was calculated as the absolute angular distance between the derived final response and the correct answer during model evaluation.

As shown in Table S3, the model reproduced the observed angular distance closely and also produced final error values comparable to the observed data, suggesting that fitting memory updating also yielded plausible final response accuracy.

Table S3. Observed versus Simulated Final Error and Angular Distance

|  | Final error Mean (SD) | RMSE | Angular distance Mean (SD) | RMSE |
| --- | --- | --- | --- | --- |
| Observation | 48.51 (51.24) | - | 43.95 (50.94) | - |
| Simulation | 44.17 (43.77) | 23.41 | 42.95 (40.22) | 27.60 |

**The Relationship Between Error Magnitudes and Final Memory Precision**

The core hypothesis of the error-driven learning (EDL) account is that larger errors drive greater memory updating, rather than directly improving final memory precision. To examine whether larger practice-test errors were associated with more precise final memory, we conducted an additional von Mises analysis of signed final-test response errors. Final-test precision was modeled using the concentration parameter κ of the von Mises distribution, with practice-test error magnitude entered as a predictor.

The results showed that larger initial errors were associated with lower final test precision (Experiment 1: *β*=−.0597, *t*=−7.40, *p*<.001; Experiment 2: *β*=−.0431, *t*=−4.58, *p*<.001). Thus, items with larger initial errors did not show more precise final memory. In Experiment 2, subjective errors were also not significantly related to final test precision (*β*_PredE_=−.0003, *t*=−0.17, *p*=.864; *β*_PercE_=−.0005, *t*=−0.07, *p*=.941).

These findings support the distinction between final memory precision and memory updating magnitude. The EDL account does not predict that initially poorly remembered items should become more precise than initially well-remembered items. Rather, it predicts that larger error signals should produce greater updating of the memory representation.
